# Male suicide among construction workers in Australia: a qualitative analysis of the major stressors precipitating death

**DOI:** 10.1186/s12889-017-4500-8

**Published:** 2017-06-19

**Authors:** Allison Milner, Humaira Maheen, Dianne Currier, Anthony D LaMontagne

**Affiliations:** 1 Centre for Health Equity, Melbourne School of Population and Global Health, The University of Melbourne, Melbourne, Australia; 2 Deakin Population Health Strategic Research Centre, School of Health & Social Development, Deakin University, Geelong, Australia; 3 Centre for Epidemiology and Biostatistics, Melbourne School of Population and Global Health, The University of Melbourne, Melbourne, Australia

**Keywords:** Suicide, Male construction workers, Life stressor, Job stress, Australia

## Abstract

**Background:**

Suicide rates among those employed in male-dominated professions such as construction are elevated compared to other occupational groups. Thus far, past research has been mainly quantitative and has been unable to identify the complex range of risk and protective factors that surround these suicides.

**Methods:**

We used a national coronial database to qualitatively study work and non-work related influences on male suicide occurring in construction workers in Australia. We randomly selected 34 cases according to specific sampling framework. Thematic analysis was used to develop a coding structure on the basis of pre-existing theories in job stress research.

**Results:**

The following themes were established on the basis of mutual consensus: mental health issues prior to death, transient working experiences (i.e., the inability to obtain steady employment), workplace injury and chronic illness, work colleagues as a source of social support, financial and legal problems, relationship breakdown and child custody issues, and substance abuse.

**Conclusion:**

Work and non-work factors were often interrelated pressures prior to death. Suicide prevention for construction workers needs to take a systematic approach, addressing work-level factors as well as helping those at-risk of suicide

**Electronic supplementary material:**

The online version of this article (doi:10.1186/s12889-017-4500-8) contains supplementary material, which is available to authorized users.

## Background

Like many other OECD countries, suicide is a leading cause of death among men in Australia [1]. Past research suggests that over half of the men who lose their lives to suicide are employed at the time of death [2]. Certain subgroups within the working population appear to be particularly vulnerable to suicide, including men employed in the construction industry [3–5]. A past meta-analysis of international studies published over the period 1979 to 2012 indicated that suicide rates among lower skilled workers in construction were 80% higher than the general working age population [3]. Worryingly, two later studies (using data from Australia and the United Kingdom) demonstrated that suicide among workers in low-skilled industries have increased substantially over time [4, 5].

Explanations for why suicide deaths are so prominent among these males have largely drawn on sociological and psychological perspectives. In the UK, Roberts et al. [5] show that the variation in suicide between occupational groups due to socio-economic factors doubled from the period 1970 to 2005. The study concluded that socio-economic conditions were a major determinant of male suicide among those in “manual occupations” (including construction) [5]. Others have focused on work-related stressors particular to construction, including the insecurity of work due to short term contracts, a fluctuating job market, long work hours, workplace bullying, and the use of alcohol and drugs in the workplace [6]. Researchers have also highlighted potential risk factors such as lack of mental health literacy and stigma [7], as well as the influence of “stoicism” as a barrier to help seeking in these traditional masculine environments [8]. As it stands however, most of these explanations are mainly speculative, as they have drawn on data collected from non-suicidal samples [6–8], rather than qualitative accounts of those who actually lost their lives to suicide.

The limited amount of qualitative research in this space is reflective of the field in general, in which research on male suicide is generally lacking. Among the small amount of studies in the area, one has focused on suicide attempts among males rather than deaths [9]. This study of 34 men who had attempted suicide revealed a number of key risk factors, including: mental health problems, unhelpful conceptions of masculinity consisting of stoic beliefs, social isolation and use of other non-social coping strategies, and life stressors [9]. Stack & Wasserman’s [10] qualitative study of the coronial records of males who had died by suicide found that loss of income, debt, legal issues and workplace conflict were proximal risk factors for suicide. Perhaps the most notable contribution is this area is the work of Scourfield [11] and Shiner [12] who conducted a “sociological autopsy” of 100 cases of suicide. Working with coronial data, these authors highlight the importance of relationship problems, work, and mental illness in male suicide cases. None of these studies have specifically focused on suicide among those who had been employed in the construction industry. The aim of this study is to deepen understandings of the risk factors associated with suicide among males in the construction industry, with a particular focus on work and employment context. As noted above, there has been a lack of qualitative research on the factors that might be underpinning the high rates of suicide among this occupational group. Based on the research cited above, we would argue that construction workers may be particularly at risk of suicide because of work and social contexts that compound life stressors and mental health issues.

## Methods

### Data source

We identified suicide cases using the National Coronial Information System (NCIS). The NCIS is an internet-based data storage and retrieval system that enables coroners, government agencies, and researchers to monitor external causes of death in Australia and to identify cases for further investigation and analysis. In every state of Australia, coroners investigate all suspected cases of suicide and produce a report that describes the circumstances and underlying cause of death. The standard format of corners’ report includes demographic information, circumstances of death and cause of death on the basis of autopsy, police report, and toxicology reports. In the study sample, we noted that the length of each coroners’ report varied from one page to 24 pages and it was dependent on the circumstances of deaths and coroners description style.

The quality and completeness of NCIS data varies between cases, particularly for the early years of the scheme (e.g., 2000), and suicide may be under-reported because of legislative and professional differences between states and between coroners [13]. In addition, there is a significant lag between a person’s death and recording in the NCIS, caused by delays of up to three years in the coronial process. We classified suicide methods according to the International Classification of Disease, 10th revision (ICD-10), codes X60–X84 [14]. Occupations were classified according to the Australian and New Zealand Standard Classification of Occupations (ANZSCO) [15].

### Sample selection

We conducted a keyword search of ‘police reports’ and ‘coronial findings of suicide cases’ occurring within the period January 2010–December 2014 to identify recent male suicide cases that mentioned work-related stressors at the time of death. The NCIS database allow comprehensive search by using Boolean or Wildcard search queries. The following individual keywords were used to identify possible cases: work, stress, bully, compensation, job insecurity, long hours, physical work, conflict, supervisor, boss, harassment, discrimination, redundancy, downsizing, or salary. We conducted a number of tests to ensure that these search terms returned all relevant cases. Each query resulted in a number of NCIS cases, and a few times, an NCIS case appeared more than one time in search queries. Once the keyword search was complete, the query results were combined, and duplicates were removed. The final list of cases consist of 1619 male cases employed at the time of death.

We then stratified these cases on the basis of occupational skill level (based on the ANZSCO) [15], and seven age groups [15–19, 20–29, 30–39…. 70 & more] to ensure an equal representation of occupational grouping and age. After the stratification, cases were selected randomly from each occupation skill level group and age group. We then inspected case reports to assess the availability of information about occupation and work history. Again, cases selected for review were selected randomly from within each of the stratified occupation and age groups.

On the basis of preliminary analysis of all occupation coding, we decided to group the cases from ANZSCO 3, 7 and 8 because of their theoretically similar working conditions in terms of being employed in the construction industry. For sample size, we aimed for at least 30 cases as there is a general consensus among qualitative researchers that a sample size to be 20–50 participants will provide adequate information for qualitative analysis [16–18]. Moreover, we also used data saturation technique [19] to inform us where to stop including more cases; which in the case of this study was 34 cases. This process of case selection presented above resulted in final sample of 14 cases from ANZSCO 3 Technicians and Trades Workers, 9 cases from ANZSCO 7 Machinery Operators and Drivers and 13 cases from ANZSCO 8 Labourers. More information on the process of selecting cases can be seen in Additional file 1.

### Data analysis

Thematic analysis is a process of encoding qualitative information under a theme and presenting the repeated information in a systematic way that increases its sensitivity and reliability in order to answer the research question [20]. Recently, a number of studies had used thematic analysis method to analysis coroners’ reports [11, 12, 21, 22] by using inductive and deductive approach. The main difference between inductive and deductive reasoning is that deductive reasoning is based on a hypothesis to test a theory whereas the inductive reasoning generates theory through interpretation of the data.

Our overall approach was similar to those described in Scourfield [11] in that we took a wider sociological perspective in the assessment of suicide among males. Within this overarching framework, we used deductive process of thematic analysis to identify, analyse, and report repeated patterns (themes) from coroners’ report [23–25]. Nvivo 11.0 was used for data analysis (QSR International Pty Ltd). To protect individual indentity, names, ages and geographical locations have been altered.

### Coding strategy

Coding of coroners’ reports was done in two stages. In the first stage, a coding structure was developed on the basis of pre-existing theories in job stress research. To increase rigour, the selected coroners’ reports were coded line by line by two independent researchers. In doing first-stage coding, both researchers noted potential new codes that could have been added to the coding structure. After the first stage of coding, the coding structure was revised on the basis of mutual consensus between the two coders. The second round of coding was done by one researcher on the basis of finalised codes, however, the final codes are reviewed and approved by both researchers. As suicide is a multidimensional phenomenon, we also allowed content to be selected in multiple codes, hence the same content can fit into more than one category. Code frequencies of each ANZSCO code (3 vs 7) and (7 vs 8), (3 vs 8) were compared to identify the similarities and differences between different them.

## Results

The sample included 34 cases of male suicide. The age range of cases can be seen in Table 1, as well as the occupational grouping of cases. Two thirds of the cases were of the age between 20 and 49 years. In terms of mental disorder (*n* = 17), depression was mentioned in 11 cases. There were two cases that mentioned a diagnosis of schizophrenia, two cases of Attention Deficit Disorder, one case of Bipolar Disorder, and one unspecified mental illness. In other cases, mental health problem was not mentioned an issue.

**Table 1:** Cases included in the qualitative analysis, by age group and occupational grouping

Age groups	Technicians and Trades Workers n (%)	Machinery Operators and Drivers n (%)	Labourers n (%)	Total
15–19	1 (100.0)	0 (0.0)	0 (0.0)	1 (100.0)
20–29	3 (37.5)	3 (37.5)	2 (25)	8 (100)
30–39	4 (50)	1 (12.5)	3 (37.5)	8 (100)
40–49	3 (42.86)	2 (28.57)	2 (28.57)	7 (100)
50–59	2 (28.57)	3 (42.8)	2 (28.5)	7 (100)
60–69	1 (33.3)	1 (33.3)	1 (33.3)	3 (100)
Total	14 (41.1)	10 (29.4)	10 (29.4)	34 (100)

In the qualitative accounts of suicide by coronial reports, we found evidence of a range of stressors recorded as being proximal to suicide, including work related stressors (e.g., work transition, workplace injury), stressors that could be either work or non-work related (e.g., work colleagues as witnesses, financial and legal issues, and substance abuse), and non-work related stressors (e.g. relationship dissatisfaction). Results show that these stressors frequently co-occurred and interacted in these men’s lives, along with other risk factors such as mental illness.

### Transient working experiences (*n* = 10)

There were notable age differences in transient work experiences. While the young had difficulties regarding the transition from school to work and finding permanent work, older workers had issues adjusting to new working environments. Arthur was a 21-year-old labourer who experienced problems in finding permanent work (although he was officially recorded as employed at the time of death in his coronial record).

*The deceased at the time of his death was between jobs, having previously been employed in various labouring-related jobs, mostly on a casual basis. These include, but are not limited to, construction, demolition, and gardening-related jobs. At the time of his death, the deceased had last worked on a building site, having been employed on a casual basis…his father attributed [the suicide] to the breakdown of his relationship and his inability to find steady employment. (Arthur, Labourer)*

Adam had issues settling into a new work place. Before that, he was working on contractual basis with different energy companies. Three days before his death by suicide, he started working as a labourer in a new work site. Adam also had a history of illicit drug use and had made suicide attempts in the past.

*Adam attended lunch with his family for his mother’s birthday. He expressed anger and frustration about his first day at work. He did not want to return to work the following day. (Adam, Labourer)*

Arthur and Adam both reported feeling anxious, unhappy and frustrated about work problems. In both these cases, the family members were aware about the presence of workplace stress in the deceased’s life. Other cases also frequently reported personal issues, which possibly hindered their ability to deal with workplace stressors. For example, Scott was a 63-year-old builder who had just been terminated from work because he was unable to meet job demands. He had multiple other stressors at the time of death, including the death of his mother, a recent relationship breakdown, financial and legal issues, and a recent workplace injury. These factors reportedly had a profound effect on his mental health and his ability to concentrate at work.

### Workplace injury (*n* = 4)

Workplace injuries were reported among a number of middle-aged and older workers. These injuries were often accompanied by another kind of severe pain or disability which limited a person’s mobility. For example, David had surgery for a hernia as a result of a work related injury. According to his wife, the deceased had been on Work benefits (duration was not mentioned). Due to ongoing financial difficulties he had to return to work. At the time of death, he was wearing his uniform which indicated that he worked that day.

*David had a history of depression following surgery for a work related injury. As a consequence of this injury he suffered chronic pain and was seeking treatment from his general practitioner and psychiatrist. (David, Tradesperson)*

Pain and limited mobility were linked to the loss of work, long term sickness absence and depression. Some of those with workplace injuries also reported treatment from psychiatrists for mental health issues. Ryan injured his back in an accident many years ago and he reinjured himself in a car accident one week before he suicided. According to his friend, he complained about his sore back the last time they spoke. He had recurrent workplace injuries which were thought to have contributed to ongoing pain and depression.

*The deceased had a sore back accident. He said he had injured it in a car accident. He reinjured it at work a week ago. (Ryan, 48, Painter).*

### Role of work colleagues (*n* = 10)

In 10 cases, work colleagues were the first ones to notice the absence of the deceased, or be the first person to find the deceased after their suicide. They also acted as important witnesses in the police reports. Sometimes the name of work colleagues were mentioned in suicide notes, indicating the level of trust and value the deceased gave to that person. For example, work colleague Michael was the first to find James, a 26 year old construction worker.

*His friend Michael came in the morning to his house to collect the deceased for work. This is a usual routine. On arrival the deceased was not waiting at the front of the residence as he usually is… Michael has walked into the premises and has located the deceased lying on the kitchen floor with a 22 rifle located beside him.* (James, Construction worker)

In another case, 24-year-old construction worker Stephen had confided in work colleague Paul about his intent to suicide just one day before his death.

*Last night I got home from work. Stephen came to my place to have a chat about him and how he was feeling. We spoke about an incident that occurred a while ago where he was talking about hanging himself with a rope that he had in his hand.* (Stephen, Construction worker)

### Financial issues (*n* = 7)

Of the financial issues, the inability to pay tax loans was reported most frequently. These tax debts reportedly had a flow on effect on employability and ability to obtain further loans. Scott was a builder and had not made a tax return for three years prior to his death. Scott also had recent life events including the death of his mother and legal issues with his former partner. Adam also had problems adjusted to a new working environment and had a history of drug use.

*The deceased was a builder and had been experiencing significant financial problems over the past 18 months as a result of tax debts, court proceedings and his business dealings. His tax debt had a substantial impact on his ability to undertake building constructions due to his inability to gain home warranty insurance. A large amount of financial and legal documentation was located in piles on the kitchen bench of the residence, suggesting the deceased had been experiencing significant financial difficulties. (Scott, Builder)*

*Adam had about $20,000 in debts, but also assets in the way of life insurance and superannuation. These assets exceeded his debts, but were not accessible to him. (Adam, Labourer)*

In another case, Johns’ rent was overdue and he feared that the real estate might take legal action against him. This was in addition to the amount he owed to the *Australian Taxation Office.* Documents related to legal and financial issues were found at the scene of death.

*There was a letter on behalf Australian Taxation office stating amount owing and documents from Centrelink. His wife further stated the deceased was overdue with his rent for the last 3 weeks and believed the real estate was taking action to recover overdue payments (John, Plasterer)*

### Legal problems (*n* = 4)

Legal problems were mentioned under a number of different circumstances including workplace legal issues, financial crisis (e.g. debt, taxation), and post-divorce legal matters. There were two cases where workplace legal issues were identified as a major stressor preceding death. In one case, the deceased suicided while he was being investigated for stolen items (e.g. rakes, top soil etc.) from work sites. The deceased had previously disclosed his concern about this investigation to his wife. Although, according to his work supervisor, other employees were also being investigated.

*His wife noticed that he seemed a bit down and asked what was going on. He showed her a letter from work that suggested he was being investigated for* ‘the use of work resources for personal gain’*. He told his wife that it was in relation to some planks of wood that he had bought home. (Cooper, Road service worker)*

Scott was a builder who had a number of legal issues prior to death. He had multiple other stressors including the death of his mother, a recent relationship breakdown, financial and legal issues, and a recent workplace injury.

*The deceased was involved in a number of civil legal proceedings involving his ex-partner and mother of his two children, and former clients of his business. (Scott, Builder)*

### Relationship breakdown and child custody issues (*n* = 18)

Marriage break up and relationship issues emerged as an important theme amongst 18 workers.

*The defacto was in a relationship with the deceased for three years and they lived together until a week ago when their relationship ended resulting in him moving out. Since moving out the deceased had been depressed and having trouble accepting that the relationship was over. (Kraig, Machinery worker)*

In another case, Hunter was a 25-year old that was upset about some offensive remarks made by his former partner on social media.

*He had split from his girlfriend that morning. He read some derogatory remarks regarding the split on Facebook. (Hunter, Truck driver)*

Separated or divorced men with young children appeared to have issues in negotiating access to their children. Francis had been recently separated from the partner and was upset being not able to meet with his children on a frequent basis. These issues were also noted in his suicide note.

*The deceased’ and his partner’s relationship was on and off for many years, with her and the children moving in and out of the home on multiple occasions. The two remained in regular contact regarding financial matters and matters over the children. Hostility remained between the two as the deceased wanted his wife to return to the family home with the children. (Francis, Forklift Driver)*

### Substance abuse (*n* = 7)

The use of substances such as heroine, methamphetamine (commonly known as “ICE”), Methylenedioxymethamphetamine (commonly known as Ecstasy), cannabis and chronic heavy drinking was noted in seven out of 34 cases. Adam was a labourer with a heroine addiction who had multiple stressors at the time of death.

*Adam had a long history of substance abuse, including illicit drugs and prescription medication. His treating general practitioner, Dr. Joe Good reported that Mr. Adam was addicted to heroin with heavy daily use. He commenced the methadone program on a few months ago, however he was observed to have great difficulty ceasing heroin use. (Adam, Labourer).*

In another instance, Francis (a forklift driver) mentioned that he has been using the drug ICE, which often caused him to be violent. He had issues with his partner and also made suicide attempts in the past.

*He was known by family members to occasionally use the drug ‘Ice’ which caused his behaviour to become erratic and violent, thus eventually leading to the breakdown of their relationship.* (*Francis, Forklift Driver)*

## Discussion

### Overview of main findings

This study highlights several important proximal risks prior to the suicide of construction workers. Many of these stressors reflected work and industry related factors, such as job insecurity, transient working conditions, issues connected to business and financial management, and fear of legal prosecution in relation to debt and conduct at work. We would argue that these work related risks reinforce the cultural norms about masculinity in the construction industry, and in combination heighten the risk of suicide among construction workers. Substance issues, alcohol use and mental health issues were other prominent factors recorded in the suicide of these workers. Our analysis also revealed family breakdown and the lack of access to children as major stressors. It appears that colleagues at work played an important role in terms of being a confidant and having regular face-to-face contact with the deceased prior to their death.

### Study limitations

As identified in Scourfield et al. [11], a problem in relying on coronial data is that it is likely to have identified only a specific range of risk factors that were obvious to those in the investigation of suicide cases, next-of-kin and other witnesses. There is also likely to be problems related to recall bias. We would also highlight that our sample will not comprehensively cover all stressors facing those employed in construction. Thus, there will be other factors (e.g., bullying) that we did not identify in this study which have been previously cited as risks for suicide [6]. Despite this limitation, our thematic analysis did identify many of the same risk factors as identified in past research. We would also acknowledge the possibility that our approach overestimates the importance of some work-related stressors. It is also important to note that all of the themes we discussed above will be interrelated and that, more than likely, it is difficult to distinguish or disentangle work from non-work related issues. For example, lack of job security, and ongoing financial issues are likely to flow over to affect relationship dynamics, and vice versa, which could be mediated by feelings of inadequacy or other pathways. Thus, risk factors combine to create a ‘perfect storm’, in which workers will be particularly vulnerable to suicide.

Another limitation is that this type of analysis is not able to adequately assess distal, chronic or long-term risks. For example, those employed in construction are often subject to low job control (characterised by the lack of ability to have a say over how and when work is conducted), high job demands and long working hours, driven by industry-related timelines. These psychosocial stressors have been identified as risk factors for a large number of common mental health problems, chronic illnesses and mortality [26–28]. Even more relevant to the current study, a recent publication using a population-level case-control design identified low control and high demands as key risk factors for suicide among employed males [29]. This corroborates past research from other countries [30–35] and highlights these as additional factors raising the risk of suicide among workers in these occupations. Similarly, coronial reports would be unlikely to explicitly highlight the circumstances of mental health problems, mental health stigma and lack of help-seeking. However, as we mention below, these are recognised risk factors in the construction industry. We would also acknowledge the fact that the study findings may lack generalizability due to its small sample size and explicit focus on construction.

### Identified risk factors for suicide among men in construction

Employment opportunities in the construction industry are closely tied to fluctuations in the national economy. The need to be responsive to wider global economic market means that the industry has a highly casualised workforce that is dependent on available contracts for ongoing work [36]. Because of this, workers in the industry are more likely to be exposed to job insecurity, which has been identified as a risk factor for mental ill-health across a number of studies [28]. In the context of our study, we would suggest that the overarching structure of the industry fuels a number of the work-related risk factors seen in our study, including job insecurity and transient working conditions, as well as feelings of pressure at work and debt.

Financial, legal, relationship, and family related stressors and were also identified in our qualitative study, as in previous studies [11, 37, 38]. Other factors identified in past research [11, 38] such as sexual jealousy and concern about the stability of housing were not apparent in our small study. Mental illness and substance use was apparent in over half of cases, supporting the argument that this is a major risk factor for suicide [39]. Although we did not have information on diagnosis in all cases, coronial reports indicated problems such as mood disorders and schizophrenia. Often there was a combination of stressors apparent in a person’s life before they died by suicide. This highlights the complex and multi-layered number of factors present in these mens’ lives before their suicide. For example, wider structural factors in the construction industry such as job insecurity are likely to combine with latent factors such as greater stigma against mental illness and help seeking to create an environment where men are at heightened risk of suicide.

### Work culture, masculinity, stigma and risk of suicide in the construction industry

Contextual factors, such as where and how people are employed, are important influences on health and normative performances of gender [40]. For example, men in highly male dominated occupations maybe more likely to display strongly held gender norms [41]. A number of studies from the 1980s and 1990s identified strongly held gender norms (e.g., restricted emotionality; restrictive affectionate behavior between men; and conflicts between work and family relations) as risk factors for depression [42] and lack of help seeking among males with respect to health problems [43]. These findings have been confirmed in later analyses [44–46]. Since this time, research has indicated that certain men are reluctant to seek professional help for mental health problems because such behaviors may not fit with beliefs about masculinity and male appropriate behaviors [40, 47–49]. The construction industry is characterised as having a culture dominated by traditionally held beliefs about masculinity with high amounts of stigma against suicide and mental health problems [8, 50]. These cultural attitudes may lead to circumstances where men feel as though they are unable to seek help in times of distress. We would suggest more research on these topics. At the same time, our analysis suggest that social support from work colleagues will be particularly important among those experiencing difficulties, which suggests the value of “mateshipness;” thus male bonds in the industry can be targeted as potential protective factors. We would argue this highlights the importance of colleagues playing a role in suicide prevention initiatives [7, 51].

## Conclusion

This study has demonstrated that there are a number of inter-related stress factors apparent prior to the suicide of construction workers; many of which are amenable to preventive intervention. There has been increased interest in the role of “gatekeepers” as people who are able to identify, provide support for, and connect those at risk of suicide with ongoing professional care [51, 52]. The finding that work colleagues appeared to play an important role in a persons’ life prior to their death suggests the potential for these people to act as gatekeepers. In addition, suicide prevention activities need to consider the wider context in which a person lives and works. Construction is prone to adverse employment conditions, which may place workers at risk of a range of mental and other health problems. Because of this, we would suggest the need for suicide prevention initiatives to take an industry-wide focus and address suicide at a primary (reduce risk factors for suicide, promote protective factors), secondary (ensuring people are supported and able to access help when they need it), and tertiary level (providing treatment for those at acute risk, and rehabilitation back into work) [53].

## Additional file

Additional file 1: Provides list of occupation and sample selection process. (DOCX 20 kb)

## Abbreviations

ANZSCO: Australian and New Zealand standard classification of occupations
ICD: International classification of disease
JHREC: Justice Human research ethics committee
MDMA: Methylenedioxymethamphetamine
NCIS: National coronial information system
OECD: Organisation for economic co-operation and development
