# Supplementary material for: Male suicide among construction workers in Australia: a qualitative analysis of the major stressors precipitating death
Source: BMC Public Health. 2017 Jun 19;17:584. doi: 10.1186/s12889-017-4500-8 (PMC5477155; doi:10.1186/s12889-017-4500-8)
Supplement: Additional file 1: — Provides list of occupation and sample selection process. (DOCX 20 kb) [file 12889_2017_4500_MOESM1_ESM.docx]

**Additional file 1.**

There are eight major ANZSCO major groups which are 1. Managers, 2. Professionals, 3.Technicians and Trades Workers, 4.Community and Personal Service Workers, 5.Clerical and Administrative Workers, 6.Sales Workers, 7.Machinery Operators and Drivers and 8.Labouers. These major categories are further divided in 1,014 occupations. We conducted a keyword search of police reports and coronial findings occurring within the period January 2010-December 2014 to identify recent male suicide cases that mentioned work-related stressors at the time of death. The following individual keywords were used to identify possible cases: work, stress, bully, compensation, job insecurity, long hours, physical work, conflict, supervisor, boss, harassment, discrimination, redundancy, downsizing, or salary. Table 1 shows the number in each ANZSCO major categories based on the keyword search.

Table 1. Overview of suicide cases by ANZSCO major occupational grouping

| ANZSCO Codes | Female | Male | Total |
| --- | --- | --- | --- |
| 1.Managers | 23 (16.2) | 119 (83.8) | 142 |
| 2.Professionals | 115 (36.9) | 197 (63.1) | 312 |
| 3.Technicians and Trade workers | 24 (5.1) | 445 (94.9) | 469 |
| 4. Community and Personal service workers | 49 (30.2) | 113 (69.8) | 162 |
| 5. Clerical and Admin staff | 58 (35.6) | 105 (64.4) | 163 |
| 6. Sales workers | 22 (22.0) | 78 (78.0) | 100 |
| 7. Machinery Operators and Drivers | 8 (2.8) | 275 (97.2) | 283 |
| 8. Labourers | 36 (11.1) | 287 (88.9) | 323 |
| Total | 335 (17.1) | 1619 (82.9) | 1,954 |

Based on the keyword search, we got 1,954 cases of them 82.8% (n=1,619) were male cases. While its consistent with the general higher trend of suicide in males, the above table is also suggestive of the elevated risk of work related suicide amongst males is higher across all occupation groups.

For this study we focused on the ANZSCO category 3, 7 and 8 which are Technicians and Trades Workers, Machinery Operators and Drivers and Labourers respectively because of their theoretically similar working conditions in terms of being employed in the construction industry.

There were 1,007 such cases (see Table 1). These cases were stratified these cases on the basis of occupational skill level (based on the Australian and New Zealand Standard Classification of Occupations)([ANZSCO, 2009](#_ENREF_2) ), and ten age groups [15-19, 20-29,…. 70 & more] to ensure an equal representation of occupational grouping and age. After the stratification, cases were selected randomly from each occupation skill level group and age group. Case reports were then inspected to assess the availability of information about occupation and work history. Again, cases selected for review were selected randomly from within each of the stratified occupation and age groups. We aimed for a sample of at least 30 cases for qualitative analysis. The process of case selection presented above resulted in final sample of 14 cases from ANZSCO 3 Technicians and Trades Workers, 9 cases from ANZSCO 7 Machinery Operators and Drivers and 13 cases from ANZSCO 8 Labourers.
